# Neoantigen-Based Immunotherapy in Lung Cancer: Advances, Challenges and Prospects

**DOI:** 10.3390/cancers17121953

**Published:** 2025-06-12

**Authors:** Xiong Li, Ya-Juan Zhu, Ying Xue, Ting-Ting Chen, Xiao-Ke Sun, Hong-Yang Shi

**Affiliations:** 1 Department of Gastroenterology, the Second Affiliated Hospital of Xi’an Jiaotong University, Xi’an 710004, China; lixiong0323@stu.xjtu.edu.cn (X.L.); 3122315382@stu.xjtu.edu.cn (Y.X.); xjtu.sxk@gmail.com (X.-K.S.); 2 State Key Laboratory of Biotherapy, Department of Biotherapy and Cancer Center, West China Hospital, Sichuan University, Chengdu 610041, China; yajuanzhu424@gmail.com; 3 Department of Gastroenterology, the Second Hosptial & Clinical Medical School, Lanzhou University, Lanzhou 730030, China; chentt2024@lzu.edu.cn; 4 Department of Respiratory and Critical Care Medicine, the Second Affiliated Hospital of Xi’an Jiaotong University, Xi’an 710004, China

**Keywords:** lung cancer, neoantigen, immunotherapy, precision medicine, clinical application

## Abstract

Lung cancer ranks among the most prevalent malignant tumors, characterized by delayed diagnosis, limited treatment efficacy, and generally poor prognosis. Consequently, there is an urgent demand for effective treatments. Neoantigen-based immunotherapy emerged as a promising strategy in lung cancer treatment as ideal targets for immunotherapy interventions. Neoantigens are tumor-specific antigens produced by genetic mutations in somatic cells, which, unlike tumor-associated antigens, are found only in tumor tissue. Neoantigen immunotherapy has the advantages of high specificity, high safety and low immunotolerance, especially for lung cancer patients with a high tumor mutation load. We systematically summarize the biological characteristics, prediction and identification, mechanism of action, clinical application, risk challenges, and prospects of neoantigens as a novel precision immunotherapy method in lung cancer to provide valuable insights and references for the advancement of lung cancer treatment strategies.

## Simple Summary

Lung cancer, a leading malignancy with delayed diagnosis and poor prognosis. Neoantigen-based immunotherapy has emerged as a precision strategy, targeting tumor-specific antigens generated by somatic mutations. Unlike tumor-associated antigens, neoantigens are exclusive to tumor cells, offering high specificity, safety, and reduced immunotolerance—particularly effective in high tumor mutation burden cases. This review synthesizes neoantigen biology, predictive methodologies, mechanisms, clinical applications, and challenges, highlighting their potential to enhance treatment efficacy. By addressing current limitations in identification, tumor heterogeneity, and therapeutic resistance, neoantigen-driven approaches aim to advance personalized immunotherapy, providing critical insights for optimizing lung cancer management and fostering future research directions.

## 1. Introduction

Lung cancer ranks as the second most common cancer and the leading cause of cancer death worldwide; according to the latest International Agency for Research on Cancer (IARC) report (2022), it accounted for 2.5 million new cases (representing 12.5% of total global cancer incidence) and 1.8 million deaths (constituting 18.4% of global cancer mortality) [1,2,3]. Lung cancer is primarily categorized into small cell lung cancer (SCLC) and non-small cell lung cancer (NSCLC), with NSCLC accounting for over 85% of all cases. Globally, adenocarcinoma has surpassed squamous cell carcinoma as the most common NSCLC subtype, largely due to its increased incidence among non-smokers and women. Although SCLC incidence is declining, it remains highly aggressive with a propensity for early metastasis and neuroendocrine features, and more than 95% of cases are linked to smoking [4,5]. Lung cancer is characterized by atypical symptoms, late diagnosis, and poor prognosis, resulting in a considerable burden on global health and economy [6,7,8]. The primary treatment of lung cancer mainly includes surgical intervention and chemoradiotherapy for early-stage lung cancer, while advanced-stage patients often benefit from targeted therapy drugs and immune checkpoint inhibitor (ICI) therapy [3]. Despite advancements in treatment regimens substantially prolonging the survival time of patients, the emergence of treatment resistance heightened the risk of recurrence. Therefore, the exploration of novel therapeutic strategies to target lung cancer is pivotal for improving the survival rate of patients.

The occurrence and development of lung cancer is a complex process. The molecular mechanism and treatment strategies for lung cancer are usually determined by multiple factors. DNA damage and repair, abnormal expression of proto-oncogenes and tumor suppressor genes, as well as aberrant DNA methylation and histone modification, often culminate in multiple gene mutations and epigenetic alterations. These factors collectively contribute to the unexpected proliferation of tumor cells, ultimately resulting in the onset of lung cancer [9]. Based on the complex molecular biological pathway of the occurrence and development of lung cancer, personalized neoantigen immunotherapy has been proposed. Neoantigens are tumor-specific antigens (TSA) derived from somatic gene mutations [10,11,12]. Unlike tumor-associated antigens (TAA), both expressed in tumor tissues and normal tissues, neoantigens are limited in tumor tissues [13]. Immunotherapy directed towards neoantigens offers distinct advantages including heightened specificity, enhanced safety profile, and reduced immune tolerance [14,15]. Furthermore, lung cancers characterized by elevated tumor mutation burdens are prone to generating abundant tumor neoantigens, thus facilitating robust anti-tumor responses in the host immune system [16,17]. Consequently, personalized targeted neoantigen therapy for individual patients emerges as a promising strategy for both research and clinical intervention in lung cancer treatment.

## 2. The Production and Immune Mechanism of Neoantigens in Lung Cancer

Tumor antigens can be produced by abnormal and over-expressed normal proteins in tumors [18,19,20]. Oncogenes or tumor suppressor genes that drive uncontrolled cell proliferation; carcinogenic viral antigen; and gene mutations that change the sequence that codes for proteins [21,22,23]. In lung cancer, tumor-specific neoantigens primarily arise through three distinct pathways: (1) somatic genomic alterations such as non-synonymous single nucleotide polymorphisms (SNPs), short insertions/deletions (indels), frameshift events, and structural rearrangements; (2) transcriptional dysregulation, including aberrant splice variants, intron retention, or premature transcriptional termination; and (3) tumor-selective post-translational modifications (PTMs) like methylation, phosphorylation, acetylation, and glycosylation. These mechanisms collectively generate novel peptide epitopes absent in normal pulmonary tissues, underpinning their immunogenic potential in NSCLC and SCLC microenvironments [24,25,26,27]. Crucially, TSA is tumor-specific and enables precise immune recognition while minimizing off-target effects, positioning TSAs as prime targets for next-generation immunotherapies.

Antigenic therapy for lung cancer mainly focused on TAAs. However, the effectiveness of targeted treatment for TAA is also hindered by central and peripheral immune tolerance mechanisms [28,29,30,31]. Despite extensive clinical evaluation in lung cancer, TAA-directed immunotherapies targeting antigens like MAGE-A3 and MUC-1 have demonstrated limited efficacy in pivotal trials [32,33]. This therapeutic challenge stems primarily from the ectopic expression of TAAs in non-malignant pulmonary tissues, which provokes on-target/off-tumor immune responses culminating in treatment-limiting autoimmune toxicities [14,31].

Therefore, the focus of antigenic therapy for lung cancer has shifted towards neoantigen-targeted anti-tumor regimens. Lung cancer can spontaneously generate somatic mutations that produce abnormal protein/peptide segments, known as neoantigens [34]. These neoantigens can directly interact with major histocompatibility complex (MHC) molecules, activating cytotoxic CD8^+^ T cells and CD4^+^ T cells to initiate an anti-tumor immune response [35,36,37]. Neoantigens released into the tumor microenvironment are captured by circulating antigen-presenting cells (APCs), particularly dendritic cells (DCs). These neoantigen-loaded APCs subsequently migrate to the tumor-draining lymph node (TDLN), where the peptide-MHC is recognized by the T cell receptor (TCR), stimulated by corresponding co-stimulatory signals and cytokines. Normally, short peptides presented on MHC-I activate CD8^+^ T cells, while long peptides presented on MHC-II activate CD4^+^ T cells [38]. The activated antigen-specific T cells undergo clonal expansion and differentiation into effector and memory T cells. These T cells migrate and infiltrate the tumor and initiate anti-tumor immune effects [30,39,40,41,42,43,44]. Since neoantigens exhibit tumor-specific expression patterns, they largely evade recognition by both peripheral and central immune tolerance mechanisms. Meanwhile, given the characteristically high tumor mutational burden observed in lung cancer, the development of personalized neoantigen-targeted immunotherapy regimens represents a groundbreaking therapeutic paradigm for lung cancer patients [45].

## 3. The Prediction and Identification of Neoantigens in Lung Cancer

**Figure 1:**
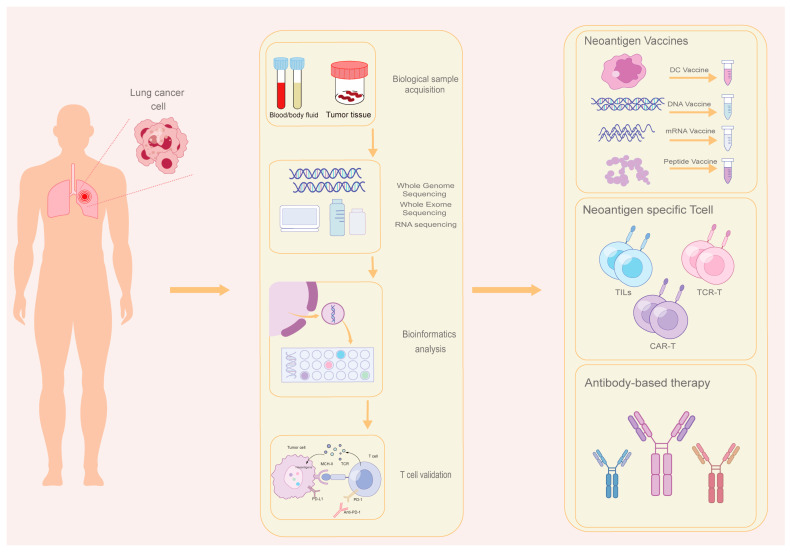
The prediction and classification of neoantigen-based therapy in lung cancer. The prediction process of individual neoantigen includes the following steps: firstly, the process begins by collecting tumor and non-tumor tissue samples from patients, followed by sequencing the extracted samples using techniques such as WGS, WES, or RNA-seq. Subsequently, bioinformatics analysis methods are employed to predict and analyze neoantigens generated by mutated genes using sequencing data. These methods involve variant calling, HLA typing, and interaction prediction. Finally, the selected neoantigens were validated by T cells. The classification of neoantigen-based therapy includes neoantigen vaccine, neoantigen-specific T-cell and antibody-based therapy. WGS: whole genome sequencing, WES: whole exome sequencing, RNA-seq: RNA sequencing, HLA: human leukocyte antigen.

Neoantigens exhibit tumor-restricted expression patterns, showing high specificity to malignant tissues with no detectable presence in normal counterparts. Their molecular configurations diverge substantially from endogenous proteins found in native tissues or cellular components. Furthermore, as neoantigens escape negative thymic selection during central tolerance development, they possess strong immunogenic foreignness that enables effective recognition and targeting by host immune surveillance systems. One crucial aspect of neoantigens is their mutational randomness. The sites and types of tumor mutations are random and irregular. Notably, neoantigens exhibit characteristics of distributional clustering. Each type of tumor cell expresses a distinct set of neoantigens, often displaying a clustering or clonal distribution pattern [30,46]. Considering these characteristics of tumor neoantigens, the generation of numerous somatic mutations and intrinsic polymorphisms in tumor hosts poses a challenge for accurately predicting and effectively screening neoantigen candidates [47]. Currently, the identification of neoantigens mainly relies on the next-generation sequencing (NGS) technology and bioinformatic pipelines [48,49]. Firstly, the process begins by collecting tumor and non-tumor tissue samples from patients, followed by sequencing the extracted samples using techniques such as whole genome sequencing (WGS), whole exome sequencing (WES), or RNA sequencing (RNA-seq). DNA sequencing allows for the identification of tumor-specific somatic mutations through comparative analysis of tumor types. Additionally, RNA sequencing aids in detecting tumor-specific somatic mutations by analyzing the mutations based on the gene expression scores obtained from RNA sequencing data, thus establishing mutation consistency. Subsequently, bioinformatics analysis methods are employed to predict and analyze neoantigens generated by mutated genes using sequencing data. These methods involve variant calling, human leukocyte antigen (HLA) typing, and interaction prediction (such as HLA-neoantigen-TCR interaction). The goal is to obtain tumor neoantigens with heightened immunogenicity, capable of inducing more efficient immune response effects within the body [50,51,52,53]. There are two mainstream identification methods for neoantigens, including computerized algorithm analysis and mass spectrometry sequencing screening predictive model analysis [54]. These approaches facilitate the identification and characterization of neoantigens, aiding in the selection of potential targets for neoantigen-based immunotherapies (Figure 1).

Computational neoantigen discovery employs algorithmic pipelines to identify non-synonymous mutations and derive resultant immunogenic epitopes through dual prediction paradigms: (1) affinity-driven modeling quantifies mutant peptide-HLA binding dynamics, leveraging evidence that high-affinity interactions potentiate antigen-specific CD8^+^ T cell priming—exemplified by Pyke et al.’s SHERPA algorithm, which integrates allele-specific immunopeptidomics to systematically rank MHC-peptide binding kinetics and epitope stability [55]; (2) surface presentation profiling simulates biophysical parameters to predict mutant epitope display probability via MHC-I complexes. SHERPA enhances validation accuracy by machine learning-based integration of affinity-presentation synergy, thereby optimizing neoantigen prioritization for lung cancer immunotherapy. However, due to the increased flexibility and complexity associated with MHC-II molecular binding epitopes, there is limited research on MHC-II molecule prediction. Notable prediction algorithm platforms for MHC-I molecules include NetChop, NetMHC, NetCTL, and NetMHCpan [56]. These platforms facilitate the precise identification of tumor neoantigens by forecasting crucial factors, such as the human proteasome cleavage site, peptide binding affinity with MHC-I molecules, and tumor abnormal protein (TAP) transport efficiency.

Mass spectrometry sequencing screening predictive modeling analysis elutes antigenic peptides from peptide-MHCs for mass spectrometry sequencing. This method narrows down the range of neoantigens and improves the accuracy of neoantigen identification [57,58,59]. However, some low-abundance peptides are easily underestimated due to the low sensitivity of this method. The combination with HLA typing information was proposed to establish a computer deep learning model [60,61,62,63]. For example, Pe et al. constructed an iConMHC prediction model based on the deep learning framework of convolutional neural networks (CNNs) to analyze the physical and chemical interaction properties between paired neopeptides and the paired amino acids in the MHC to predict the peptides. Based on the interaction properties, this method predicts the binding affinity between peptide antigens and specific MHC genes, thus improving the sensitivity and accuracy to identify neoantigens [35].

With the development of bioinformatics, emerging methods have been applied in the identification process of neoantigens [64,65,66,67]. Tools like pVACtools integrate mutant peptide data from various sources, offering a comprehensive solution for tumor neoantigen prediction [68]. TIMiner facilitates the analysis of RNA-Seq data and somatic DNA mutations within a single sample [69]. Emerging computational tools, including T cell antigen screening platforms, signaling and antigen-presenting bifunctional receptors (SABRs) leveraging TCR-pMHC interactions, and MHC-TCR chimeric receptors (MCRs), address specific limitations in neoantigen prediction, particularly TCR-pMHC binding dynamics and MHC class II epitope identification [70,71]. Other prediction tools like MuPeXI, TSNAD, Open Vax, pTuneos, ScanNeo also contribute to this field [72,73,74,75,76]. At present, these bioinformatics-based prediction tools are still in the developmental phase, and their application in clinical research needs to be further explored.

Some of the predicted and screened neoantigens did not show significant anti-tumor effects in clinical trials, which can be attributed to the immunogenicity of these neoantigens. Therefore, it is essential to evaluate and verify the immunogenicity of neoantigens before applying them in clinical studies to ensure more efficient tumor targeting [77]. Previous studies have proposed various ex vivo experiments to validate the immunogenicity and immunoreactivity of neoantigens, such as ELISPOT, tetramer, and other cellular immune response-related assays [56,58,78,79]. These experiments assess the ability of neoantigens to elicit an immune response. However, accurately predicting neoantigens still poses a challenge due to factors like HLA polymorphism, variations in expression abundance and affinity of MHC-I molecules, and the limited accuracy of MHC-II class molecule prediction [80,81,82,83,84]. There is a lack of high-throughput, effective, and rapid methods for the identification and verification of neoantigens, as well as a lack of standardized protocols. Therefore, to address these issues, it is crucial to establish and expand a comprehensive database of real neoantigens. Analyzing the characteristics of these real neoantigens can help improve the accuracy of prediction algorithms and enhance screening efficiency. Establishing standardized identification and verification procedures for neoantigens in lung cancer will be a new direction for future exploration and improvement.

## 4. Application of Neoantigen-Based Therapy in Lung Cancer

**Figure 2:**
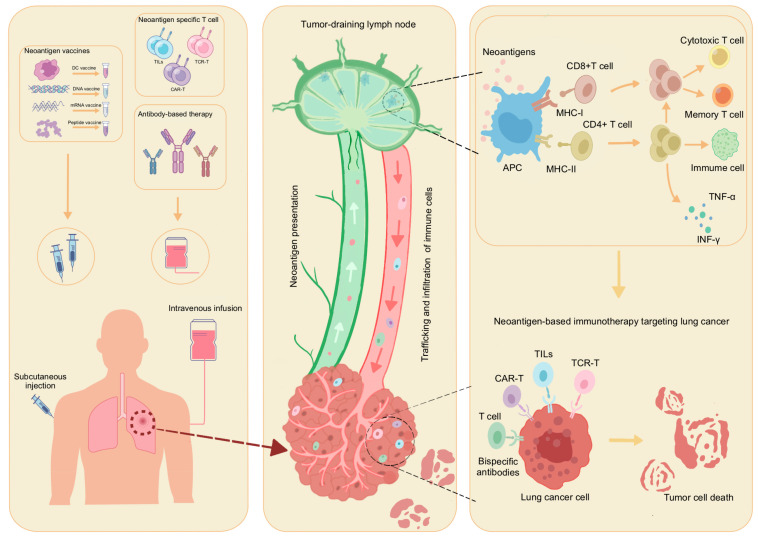
The mechanism and effect of neoantigen-specific T cells in lung cancer. Neoantigens are cancer-specific abnormal peptides recognized as non-self entities that induce host immune responses. Upon release into the tumor microenvironment, these neoantigens are captured by circulating APCs), such as dendritic cells, which subsequently migrate to TDLNs. Within TDLNs, neoantigens form complexes with MHC molecules and receive co-stimulatory signals and cytokine stimulation. This process activates antigen-specific T cells, triggering their clonal expansion and differentiation into effector subsets. The expanded T cell populations then infiltrate tumor tissues, initiating anti-tumor immune responses that eliminate lung cancer cells through cytotoxic activity. APC: antigen-presenting cell, TDLN: tumor-draining lymph node, MHC: major histocompatibility complex.

Immunotherapy for lung cancer stimulates and enhances the body’s immune system to recognize lung cancer-related antigens and attack tumor cells to inhibit tumor growth. Immunotherapy centered on tumor neoantigens represents a personalized treatment approach that holds promise in enhancing specific anti-tumor immune responses while minimizing or circumventing the risk of off-target effects. This approach introduces tumor neoantigens into the patient’s body through various modalities, triggering the body’s immune response, facilitating antigen–antibody interactions, stimulating specific anti-tumor immune responses, and fostering the generation of immune system memory mechanisms to eradicate tumor (Figure 2) [26,85,86,87,88,89].

Previous studies have described three primary facets of neoantigen therapy development. First, neoantigens play a pivotal role in the direct engagement of T cells in killing tumor cells, thereby serving as a cornerstone in the immunotherapeutic process [90,91]. Second, neoantigens directly activate tumor-specific T cells through MHC-mediated antigen presentation, thereby reactivating residual cytotoxic T lymphocytes (CTLs) and blocking tumor immune evasion. This mechanism complements PD-1/PD-L1 blockade (which reverses T cell exhaustion), and their synergistic combination potently enhances anti-tumor immunity. Additionally, the neoantigen burden is positively correlated with the therapeutic efficacy [9,92]. Third, neoantigen vaccines, adoptive cell therapy (ACT), and neoantigen-based antibody therapy have demonstrated safety in treating advanced tumors with their combination with other modalities or as adjuvant therapy, showing no increased adverse reactions [93,94]. The development of neoantigen immunotherapy has opened a new chapter in tumor personalized immunotherapy, enabling patients’ immune cells, such as T cells, to accurately track and attack tumor cells. Various approaches, such as the neoantigen vaccine, ACT, and neoantigen-based antibody therapy, have achieved certain clinical effects in the field of lung cancer treatment, showing promising application prospects [14,29,95]. Therefore, neoantigen immunotherapy, whether applied alone or in combination with other therapies, occupies a very important position in the precision treatment strategy of lung cancer [96].

### 4.1. The Use of Neoantigen-Based Therapy in the Lung Cancer

#### 4.1.1. Personalized Neoantigen Vaccine

Different from vaccines utilizing tumor-associated antigens, lung cancer-specific neoantigen vaccines aim to kill tumor cells via tumor antigen-specific cellular immune responses with high tumor specificity and low targeted autotoxicity. Neoantigen vaccines mainly include peptide/protein vaccines, DC vaccines, nucleic acid vaccines and other types of vaccines, which can activate anti-tumor responses of the body through different pathways (Table 1) [50,65,97,98].

Peptide/protein vaccines are the most widely used vaccines in lung cancer treatment, particularly in the NSCLC. Peptide vaccines can directly bind to MHC molecules without the APC presentation process. This direct binding activates CD8^+^ T cells, promoting robust cytotoxic responses to target tumor cells [98,99]. Notably, peptide vaccines are categorized into long peptides (15–31 amino acids in length) and short peptides (8–10 amino acids in length), each tailored to bind directly to MHC-I molecules, thereby eliciting specific T cell responses. Meanwhile, protein vaccines, rich in antigenic epitope information, surpass peptide vaccines in immunogenicity and stability, capable of eliciting both CD8^+^ and CD4^+^ T cell responses [100,101,102,103]. Nakatsura’s team reported a promising antigenic peptide vaccine targeting NSCLC patients with EGFR T790M/C797S mutations. The EGFR T790M/C797S mutation neoantigen vaccine emerges as a preferred regimen for EGFR-TKI-resistant NSCLC patients [104]. In addition, Takayama et al. demonstrated that personalized neoantigen peptide vaccines significantly improved progression-free survival (PFS) and overall survival (OS) in patients with advanced NSCLC who developed humoral immune responses to the vaccine [105]. This indicates the potential of neoantigen peptide vaccines in augmenting anti-tumor immune effects, particularly in patients with humoral immune responses. However, despite their promise, peptide/protein vaccines face challenges. Their lengthy production cycle, unique peptide epitopes, low molecular weight, susceptibility to degradation, and short half-life present practical limitations in their application [106,107]. Addressing these challenges remains pivotal in unlocking the full therapeutic potential of peptide/protein vaccines in lung cancer treatment.

DC vaccines are designed to present tumor antigens to immune cells, stimulate lymphocytes to recognize those antigens, and then target kill tumor cells that express those antigens [94,108,109,110]. In mouse NSCLC, Sun et al. estimated mutation-associated new epitopes of ASB-XIV by whole exome sequencing and RNA sequencing. Prophylactic mPhf3-DC vaccination, based on the mutated PHF3 peptide (mPHF3), can suppress ASB-XIV tumor progression by eliciting mPHF3-specific CD8^+^ T cell responses within the local tumor microenvironment, which mediate anti-tumor immunity. Furthermore, this vaccine enhanced anti-PD-1 efficacy, overcoming resistance in PD-1/PD-L1 blocker-resistant LLC1 tumors in the same model [111]. A Phase I clinical study in patients with post-operative NSCLC showed that the individual neoantigen DC vaccine was feasible in 6 out of 10 enrolled patients, with toxicity limited to grade 1–2 adverse events. In addition, systemic T cell responses were observed in 5 out of 6 vaccinated patients. T cell responses were still detectable 19 months after vaccination, demonstrating the feasibility, safety, and immunogenicity of DC neoantigen vaccines in patients with NSCLC after surgical treatment [112]. DCs, potent antigen-presenting cells, can be loaded with neoantigens by a variety of techniques, including the complete mRNA pulse, synthetic peptide pulse, and autologous whole tumor lysate (WTL) pulse as well as the pulse of fusion with tumor cells [29]. In patients with advanced NSCLC, personalized neoantigen-pulsed DC vaccines have shown promising outcomes with objective response rates (25%), disease control rates (75%), and improvements in progression-free survival and overall survival (5.5 months). The Neo-DCVac is a feasible and safe anti-tumor regimen capable of triggering specific T cell immunity and therapeutic benefit [3]. Another research team, by combining L82 pulse DC vaccination with anti-CD38 antibody treatment, effectively suppressed tumor growth through a mechanism that relies on the reduction in regulatory T cells in tumors, thereby stimulating anti-tumor immunity in a mouse lung cancer cell line LLC1 that is resistant to immune checkpoint therapy [113]. Despite the fact that the DC vaccine has become the most important method in the active immunotherapy strategy, it still faces difficulties in the application of the complicated production process, expensive production cost, and easy to cause vascular damage, electrolyte disturbance, and other adverse reactions.

Nucleic acid vaccines are mainly in the form of DNA and mRNA [67,114,115]. DNA vaccines offer advantages of easy production, stable formulation, and the ability to encode all epitopes [116]. Moreover, DNA vaccines possess intrinsic adjuvant effect, spontaneously stimulating T cell anti-tumor responses and inhibiting tumor growth. Malekshahi et al. observed that a plasmid DNA vaccine carrying carcinoembryonic antigen (CEA) stimulated the production of CEA-specific T cells. Meanwhile, the serum levels of anti-CEA-specific IgG antibodies in the tumor-bearing mice were also elevated. The secretion of interferon-γ (IFN-γ) interleukin-2 (IL-2) also stimulated T helper cell-1, contributing anti-tumor immune effects [117]. Similarly, GNOS-PV02, a personalized cancer DNA vaccine, can encode up to 40 patient-specific neoantigens and effectively activate tumor-specific T cells and generate specific anti-tumor responses in patients with advanced hepatocellular carcinoma [118]. However, DNA vaccines also have the potential risk of insertion mutagenesis and low DNA transfection efficiency.

Multiple studies have also confirmed that mRNA vaccines can safely elicit neoepitope-specific T cell responses and effectively stimulate anti-tumor immune responses, while demonstrating distinct advantages of high efficacy, low incidence of adverse reactions, and reduced costs [119]. Compared to DNA vaccines, mRNA vaccines eliminate concerns regarding the risks of insertional mutagenesis or aberrant transcription. They also circumvent the risk of side effects caused by the conversion of DNA into protein and the reduction in biodegradable components of DNA [120,121,122]. Advanced technologies in RNA synthesis, cryogenic/ambient preservation, and delivery vectors have provided a good platform for mRNA vaccine development by improving and strengthening the immunogenicity of mRNAs [116]. Oosting et al. reported that FRAME-001 is a feasible personalized vaccine formulation for the treatment of stage III-IV NSCLC. A self-amplifying mRNA neoantigen vaccine in combination with nivolumab and ipilimumab induced a long-term neoantigen-specific CD8^+^ T cell response and initiated an anti-tumor immune response in NSCLC [123]. Furthermore, Sahin et al. also demonstrated that the combination of a neoantigen-containing mRNA vaccine with anti-PD-1 (pembrolizumab) treatment induced both CD4^+^ T cell and CD8^+^ T cell responses [124]. Overall, both DC vaccines and DNA vaccines hold promise as immunotherapeutic approaches for the treatment of NSCLC, with the potential to induce specific T cell immunity and provide therapeutic benefits.

Importantly, various types of vaccines are being explored in cancer immunotherapy, including shared neoantigen vaccines, viral vector vaccines, and fusion protein vaccines [99]. For instance, a personalized vaccine (PEV) regimen involving GAd-PEV priming and MVA-PEV enhancement has been integrated with anti-PD-1 immunotherapy for the treatment of stage IV NSCLC [125]. Additionally, clinical trials are underway to evaluate the immunogenicity of shared neoantigen vaccines such as GRT-C903 and GRT-R904 in combination with nivolumab and ipilimumab for metastatic or advanced NSCLC [126]. Despite these, vaccine modalities are still undergoing research and testing phases, and they hold promise as potential additions to future tumor immunotherapy protocols.

#### 4.1.2. Adoptive Cell Therapy Based on Neoantigens

Adoptive cell immunotherapy involves the extraction, activation, or genetic modification of autologous or allogeneic immune cells in vitro. These cells that are bolstered in numbers and armed with anti-tumor properties are reintroduced into patients to enhance their cellular immune function, thereby bolstering the anti-tumor response. Adoptive immune cells play a pivotal role in regulating and augmenting the immune function of tumor patients, effectively countering the tumor’s immune evasion mechanisms. Unlike traditional therapies, cellular adoptive immunotherapy not only directly targets tumors but also harnesses the body’s immune function to suppress tumor growth, maintaining a relative dynamic equilibrium within the unique tumor microenvironment to impede progression. It boasts advantages such as potent efficacy, low toxicity, precise targeting, and the induction of memory immunity. Common treatment modalities include tumor-infiltrating lymphocytes (TIL), T cell receptor-modified T cells (TCR-T), and chimeric antigen receptor-modified T cells (CAR-T) [127]. Komuro et al. demonstrated that CD8^+^ TILs frequently inhabit NSCLC lesions. Using single-flux sequencing, they reconstituted tumor antigen-specific T cells, focusing on phenotypically depleted CD8^+^ TILs. This approach aids in identifying tumor neoantigens and elucidating the characteristics of antigen-specific T cells, thus pinpointing promising immunotherapy targets for NSCLC patients [128]. Moreover, a meta-analysis by Zhen et al. revealed that combining ACT with radiotherapy and chemotherapy significantly improves 2-year progression-free survival (PFS) (OR = 2.20, 95% CI: 1.44~3.36, *p* = 0.0003) and 2-year overall survival (*p* < 0.00001) in NSCLC patients compared to radiotherapy and chemotherapy alone [129]. In a phase I clinical trial, Xia’s research team investigated the adoptive treatment of NSCLC patients expressing NY-ESO-1. The utilization of antigenic peptides, alongside genetically engineered TCR-T cells targeting the NY-ESO-1 antigen, yielded promising results, where initially evidenced by a partial response observed in a female lung adenocarcinoma patient [130]. Notably, the treatment was well tolerated, without serious toxic adverse reactions reported. These studies collectively underscore the promising potential of adoptive cell immunotherapy in lung cancer treatment.

#### 4.1.3. Neoantigen-Based Antibody Therapy

Neoantigen-based antibody therapy represents a crucial aspect of neoantigen immunotherapy. The primary strategy involves converting TCR mimics (TCRm) antibodies or mutation-associated neoantigen (MANA)-specific antibodies into various therapeutic forms such as full-length antibodies, antibody-drug conjugates (ADCs), and bispecific antibodies (BsAbs). These antibodies target the p-MHC to identify intracellular neoantigens, thereby activating the specific anti-tumor response of the immune system [29,131,132]. Douglass et al. employed phage display methods to create mutated RAS peptide-HLA complex-specific single-stranded variable fragments (scFvs). These scFvs were then combined with anti-CD3 scFv to generate RAS/CD3 bispecial single-stranded disomes (scDbs). In vitro, studies demonstrated that these constructs induced T cell activation, leading to the subsequent elimination of tumor cells expressing mutated RAS peptide-HLA complexes [133]. Similarly, Hsiue utilized large phage library screening to identify an H2 antibody fragment with enhanced affinity to the HLA-A* 02:01-restricted p53 R175H neoantigen. By fusing this TCRm antibody fragment with a CD3-specific antibody fragment, they developed a BsAb capable of enhancing T cell activity against cancer cells and grafts expressing the p53 R175H pMHC in animal models [131]. These innovative approaches highlight the potential of neoantigen-based antibody therapy in augmenting the immune response against specific tumor antigens, thereby offering promising prospects in cancer immunotherapy.

#### 4.1.4. Clinical Trials of Neoantigen-Based Therapies

While the majority of neoantigen therapy trials in lung cancer remain ongoing, a limited number have reached completion, offering initial insights into safety, feasibility, and immunogenicity. The clinical trials focused on neoantigen—targeted therapy in lung cancer are presented in Table 2, and the key findings derived from the completed studies are summarized as follows: the NCT02897765 trial demonstrated that the personalized neoantigen DNA vaccine NEO-PV-01, combined with anti-PD-1 therapy, was feasible and safe. This regimen successfully generated new neoantigen-specific CD4^+^ and CD8^+^ T cells exhibiting a cytotoxic phenotype. Critically, these induced T cells demonstrated the capacity to traffic to tumor sites and mediate tumor cell killing [134]. Similarly, the NCT03953235 trial and NCT03639714 trail evaluated a shared neoantigen mRNA vaccine strategy (GRT-C903 targeting KRAS mutations and GRT-R904 targeting TP53/PTEN/KRAS mutations) combined with nivolumab and ipilimumab. This combination was well-tolerated in NSCLC patients and elicited measurable neoantigen-specific CD8^+^ T cell responses. Furthermore, an increased frequency of vaccine-induced T cell receptor (TCR) clonotypes was detected within treated tumor samples, providing direct evidence for vaccine-induced T cell infiltrations [126,135].

Collectively, these early results—though derived from relatively small phase I/II patient cohorts—provide crucial proof-of-concept evidence supporting the safety, immunogenicity, and potential clinical utility of neoantigen vaccines in lung cancer. The demonstrated ability to generate functional, tumor-infiltrating neoantigen-specific T cells using diverse vaccine platforms paves the way for the numerous ongoing, larger-scale efficacy trials. These subsequent studies aim to establish definitive clinical benefits and further optimize combination strategies with checkpoint inhibitors and other modalities.

### 4.2. Combination Therapy

Neoantigen-based therapy primarily operates by stimulating cytotoxic T cells to eliminate tumors. However, its effectiveness is hampered by the diverse neoantigen profiles, evolving tumor immune escape mechanisms, and the immune suppression within the tumor microenvironment [136]. Consequently, the efficacy of single immunotherapy in advanced cancer patients is often suboptimal. To improve the effectiveness of neoantigen-based therapies in lung cancer, the combination with diverse mechanisms of action was explored. This approach aims to augment anti-cancer efficacy and endure therapeutic outcomes [137,138,139].

#### 4.2.1. Neoantigen Vaccine Combined with Adoptive Cell Therapy

Recent studies have revealed the promising potential of neoantigen vaccination, demonstrating its ability to bolster the population of circulating neoantigen-reactive T cells. Moreover, these vaccinations have been shown to elicit neonatal T cell responses, overcoming the challenge of insufficient recognition of novel epitopes by T cells, often caused by inadequate cross-presentation of neoantigens by tumor cells [29,127,140]. Furthermore, certain neoantigen vaccines have been observed to shield newly activated antigen-reactive T cells from immune checkpoint signaling or FASL-mediated apoptosis, thus enabling these T cells to infiltrate the immunosuppressive tumor microenvironment (TME) and induce sustained regression of epithelial malignancies [141].

For instance, Sun et al. implemented a treatment regimen combining ACT with DC vaccination, yielding promising outcomes in the treatment of advanced lung cancer patients. Using second-generation sequencing technology, the research team identified highly immunogenic neoantigens specific to lung cancer. They then formulated DC vaccines loaded with these lung cancer neoantigens and administered them subcutaneously into lymphatic-rich regions, such as the axillary lymph node area. This approach not only effectively primed CD8^+^ and CD4^+^ T cells within the body but also facilitated the ex vivo expansion of these reactive T lymphocytes, which were subsequently reintroduced into the patient’s system to efficiently target and eliminate tumor cells [111].

#### 4.2.2. Neoantigen Immunotherapy Combined with ICIs

ICIs has emerged as a relatively effective and durable treatment strategy in a variety of malignant tumors such as NCSLC and melanoma [67,142,143,144]. However, a subset of patients failed to respond to ICIs therapy due to the absence of tumor-specific effector T cells, resulting in only limited sustained anti-tumor responses to monotherapy. Consequently, several studies have explored the combination of ICIs with neoantigen-based immunotherapy to augment tumor-reactive T cell responses and achieve enhanced anti-tumor effects [29,145].

Tumor therapeutic vaccines can induce the production of anti-tumor specific T cells, thereby furnishing additional resources for ICIs like PD-1 monoclonal antibodies and exerting a synergistic anti-tumor effect [66]. Reversely, ICIs treatment can also further enhance the anti-tumor effect of CTL. TILs are limited in tumor cells and show irreversible low reactivity due to the immune inhibitory microenvironment. In patients responding to PD-1 inhibitors, neoantigen immunotherapy can induce the expansion of PD-1^+^ CD8^+^ T cells, resulting in a temporary increase in PD-1^+^ CD8^+^ T cell circulation and the infiltration of effector T cells at the tumor site. These results indicate that ICIs can promote neoantigen-reactive lymphocyte penetration into tumors and enhance the effect of anti-tumor therapy [146,147,148,149,150,151]. Furthermore, ICIs can significantly remodel the tumor-suppressing microenvironment of neoantigen-activated T lymphocytes, reversing T cell immune tolerance, and rejuvenating depleted neoantigen-specific T cells. Therefore, the combination of these two modalities synergistically amplifies the immune response. In a clinical trial, a neoantigen mRNA vaccine combined with a PD-1 inhibitor (pembrolizumab) was used in 20 patients with unresectable advanced solid tumors (including 7 NSCLC). Five cases of partial response (PR) (including two patients previously treated with ICIs) and six cases of stable disease (SD) were observed along with a detected neoantigen-specific CD8^+^ T cell response in these patients [152]. Similarly, as first-line treatment for advanced NSCLC, the combination of the personalized neoantigen vaccine NEO-PV-01 with pemetrexed, carboplatin, and pembrolizumab induced neoantigen-specific CD4^+^ and CD8^+^ T cell anti-tumor responses without treatment-related serious adverse events [153]. In advanced squamous cell carcinoma, DC-based neoantigen vaccine combined with ICIs could produce beneficial clinical effects in PD-1 blockade-resistant lung cancer patients [154]. Clinical trials, such as NCT03633110, evaluate the safety, dose, immunogenicity, and early clinical activity of personalized cancer neoantigen vaccines GRT-C901 and GRT-R902 in combination with nivolumab and ipilimumab in patients with metastatic NSCLC. Another trial, NCT04267237 assesses the efficacy, safety, pharmacokinetics, immunogenicity, and biomarkers of the RO7198457 vaccine in combination with atezolizumab for patients with stage II-III NSCLC who are positive for circulating tumor DNA (ctDNA) after surgical resection and receiving standard of care-assisted biplatinum chemotherapy. In conclusion, the synergistic treatment of neoantigens and ICIs significantly enhances the therapeutic outcomes for lung cancer, offering a novel and promising approach to its management. Currently, numerous clinical trials are underway to delve deeper into the safety and efficacy of this combined therapy, with the ultimate goal of integrating neoantigen immunotherapy with ICIs into routine clinical practice.

#### 4.2.3. Neoantigen Immunotherapy Combined with Conventional Therapy

Traditionally, lung cancer treatment mainly includes chemotherapy, radiotherapy, targeted therapy, and surgery. The development of neoantigen immunotherapy has opened new possibilities for personalized cancer treatment. Several clinical trials have confirmed the efficacy of combining personalized neoantigen immunotherapy with traditional treatments [139]. When combined with chemotherapy, personalized neoantigen immunotherapy can reduce the dosage of chemotherapy drugs and the incidence of adverse reactions, thereby improving the quality of life of patients. Similarly, radiation therapy can enhance the expression of MHC-I on the surface of tumor cells, which in turn increases the presentation of polypeptides and killing of neoantigen-specific CD8^+^ T cells. When combined with targeted drugs, this approach can effectively reduce the tumor load and intratumoral pressure, thus enhancing anti-tumor efficacy [155,156,157,158,159]. In a study conducted by Lin’s research team, they combined LLCvac, a neoantigen vaccine based on personalized tumor DNA mutation, with bevacizumab and anti-PD-1 antibodies for the treatment of NSCLC. The results showed that this combination regimen played a stronger anti-tumor effect with an obvious reduction in tumor volume but without significant toxicity. The assessment of the tumor immune microenvironment revealed a substantial increase in the proportion of neoantigen-specific T cells in the blood with the combination therapy. Moreover, a significant infiltration of neoantigen-specific Ki67-positive CD8^+^ T cells was observed in the tumor tissue, indicating effective killing of the corresponding tumor cells [160]. These studies highlight the promising potential of personalized neoantigen immunotherapy in enhancing the effectiveness of lung cancer treatment. By combining neoantigen immunotherapy with traditional therapies, we can improve patient outcomes and pave the way for more targeted and tailored approaches to cancer treatment.

## 5. Challenges of Neoantigen Immunotherapy in Lung Cancer

### 5.1. Tumor Heterogeneity and Patient HLA Heterogeneity

Tumor heterogeneity and patient HLA heterogeneity pose significant challenges in the diagnosis and treatment of lung cancer [161,162]. Tumor heterogeneity manifests in temporal and spatial dimensions. Temporal heterogeneity refers to the variation in phenotypes and characteristics of lung cancer lesion at different stages of development. Spatial heterogeneity encompasses differences between various tumor types, within the same tumor across different patients, between the primary tumor and the metastasis, among different primary tumors in the same organ tissue, and even within cells within the tumor itself. These diverse tumor heterogeneities make it exceedingly difficult to achieve comprehensive coverage of tumor cells with neoantigen immunotherapy [163,164,165]. Consequently, the efficacy of tumor immunotherapy is substantially limited, as neoantigen prediction only encompasses a fraction of tumor types, thereby heightening the uncertainty surrounding the efficacy of neoantigen tumor vaccines [166,167]. Moreover, patient HLA heterogeneity arises from the high polymorphism of MHC molecules and significant individual differences [168]. This diversity not only complicates the screening and identification of neoantigens but also leads to the wide variation in affinity and reactivity of different individuals to tumor antigens. Such variations directly influence the generation and intensity of anti-tumor immune responses [169,170,171]. Therefore, conducting clinical studies that address tumor heterogeneity and HLA heterogeneity is imperative to lay the groundwork for the safe and effective development of neoantigen immunotherapy. These efforts are crucial for ushering in a new era of personalized neoantigen precision therapy [82].

### 5.2. Dilemmas to Predict and Identify Neoantigens

Tumor antigen prediction and identification are pivotal steps preceding the development and utilization of anti-tumor vaccines. Contemporary neoantigen prediction is predominantly enabled by NGS technologies and bioinformatic platforms, which are essential for identifying somatic mutations, determining HLA types, and forecasting potential immunogenic epitopes. Although the standards of these prediction methods vary, resulting in differences in prediction accuracy, several developed high-performance computational approaches exhibit robust predictive capabilities. For example, pMTnet—a deep learning-based model leveraging transfer learning for TCR-pMHC binding specificity prediction—achieved an AUC of 0.827 and AUPR of 0.566 on an independent test set of 619 experimentally validated TCR-pMHC pairs, outperforming conventional tools in genomic-scale analyses16. Similarly, harmonized machine learning classifiers (e.g., voting classifiers integrating logistic regression and XGBoost) trained on the NCI cohort improved immunogenic neoantigen ranking by 11.8–30% across multiple datasets (NCI, TESLA, HiTIDE) through feature engineering incorporating TCR hydrophobicity patterns and natural HLA presentation likelihood via ipMSDB [172,173]. Despite these advances, prediction accuracy remains constrained by multiple factors. Gene fusion, deletion, and insertion events further destabilize neoantigen prediction outcomes [166,174,175,176]. Additionally, the dominance of MHC class I molecule epitopes in current antigenic epitopes hampers the prediction of MHC class II molecule neoantigens, thereby limiting the accuracy of tumor neoantigen prediction algorithms. Although tens of thousands of mutations can be detected in tumor samples, neoantigen prediction typically yields only a small number of results. The results of clinical trials show that less than 1% of the total number of mutations can really stimulate the body’s specific immune response [77]. Therefore, only a fraction of valuable neoantigens is synthesized in vitro and commercially available for inducing immune responses in the clinical setting. Moreover, research suggests that CD8^+^ T cells recognize peptides of 8–11 amino acids from MHC-I, while CD4^+^ T cells recognize peptides of 12–15 amino acids bound to MHC-II [177,178]. Thus, accurately predicting the selection of neoantigens with appropriate length and number to optimize CD8^+^ and CD4^+^ T cell responses poses an additional challenge in achieving effective tumor immunotherapy.

### 5.3. Immune Escape and Immune Interference

The tumor microenvironment, along with the tumor itself, constitutes a complex tumor ecosystem characterized by heterogeneity, inhibition, relative independence, and comprehensiveness [29,179,180]. During tumor development, an ongoing battle unfolds between the tumor and the immune system. The immune inhibitory environment within the tumor microenvironment hampers the entry of immune cells and impedes the normal functioning of infiltrated immune cells [181]. This creates favorable conditions for tumor cell growth and immune evasion. Various components, including inhibitory molecules (such as PD-L1), inhibitory cells (such as Treg cells), and inhibitory cytokines (such as IL-10, TGF-β), contribute to this immune inhibitory environment. Moreover, tumor cells usually downregulate MHC-I, effectively evading immune surveillance. This downregulation results in defects in antigen presentation and hinders the activation of tumor antigen-specific CD8^+^ T cells [170,182,183,184,185]. A study using RNAseq and pathological tumor infiltrating lymphocyte assessment analyzed 258 regions in 88 early untreated NSCLC patients and found that regions with low lymphocyte infiltration showed the emergency of immunoediting. During tumor evolution, immune editing may diminish neoantigen production or cause loss of clonal neoantigen copy number, resulting in neoantigen depletion and impaired antigen presentation. In addition, tumor regions infiltrated by immune cells showed sustained immunoediting, resulting in loss of heterozygosity of HLA or depletion of expressed neoantigens, further failing the neoantigen presentation process. Therefore, this study suggests that the immune microenvironment exerts a strong selective force in early, untreated NSCLC, generating multiple immune escape pathways, and that neoantigens are edited to mediate immune escape during the evolution of lung cancer [186]. Additionally, the presence of immune interference should be considered. Studies have found that HLA Class II negative tumors can indirectly activate tumor-specific immunosuppressive T regulatory (Treg) cells, thereby leading to immunosuppression [187]. The appearance of such immune interference may offset the expected high immunogenicity of extreme mutant tumors. Therefore, a new research direction in neoantigen immunotherapy involves addressing the challenges posed by the tumor microenvironment and immune interference. Selecting neoantigen vaccines that can surmount these obstacles and positively impact anti-tumor responses is crucial for advancing neoantigen-based immunotherapy [188].

### 5.4. Tumor Associated Antigens and Public Neoantigens

TAA exhibits expression in certain normal tissues. Consequently, TAA-targeted immunotherapy risks activating immune responses in non-target tissues, potentially triggering severe auto-immune toxicity or even fatal outcomes. Furthermore, the effectiveness of TAA-based vaccines is limited by central and peripheral immune tolerance; thymic selection results in TCRs having significantly lower affinity for TAA than for neoantigens [10,14]. In contrast, neoantigens allow immunotherapies to bypass central T cell tolerance to self-epitopes, enhancing tumor-specific immunity [189,190].

Neoantigen-based immunotherapy represents a highly personalized treatment approach. Since mutations occur randomly, neoantigens generated from each individual and each tumor mutation are virtually unique. Personalized neoantigens are tailored to individual tumor mutation profiles, enabling the development of patient-specific therapeutic regimens. However, the advantage of high specificity is accompanied by the challenges of prolonged preparation time and high production cost, significantly limiting their clinical applications [191]. To address these constraints, shared neoantigens have emerged as a compelling therapeutic focus. Relative to patient-specific neoantigens, therapies utilizing public neoantigens offer dual advantages: reduced resource expenditure and production timelines, alongside mitigation of constraints inherent to personalized approaches. These features collectively enhance diagnostic and therapeutic precision in oncology. Paradoxically, the likelihood of discovering universally applicable neoantigens across patient populations remains exceptionally low, with a reported frequency below 0.005%. Consequently, developing broadly effective interventions based solely on public neoantigens presents substantial challenges [192,193]. This limitation underscores the strategic value of integrating public and patient-specific neoantigens as a promising approach for precision immunotherapy targeting common oncogenic alterations in lung cancer [194,195].

### 5.5. Production Cycle and Cost of Neoantigen Vaccine

Neoantigen tumor vaccines are highly personalized with the preparation process, spanning tumor specimen collection, sequencing, and bioinformatics analysis. It usually takes several months to vaccine preparation. This timeline is further prolonged by the requisite extensive animal models and well-controlled clinical trials needed to validate the safety and efficacy of the tumor. Consequently, many patients with advanced tumors do not receive timely treatment with neoantigen vaccines. Shortening the preparation cycle of personalized neoantigen-based tumor vaccines is thus an urgent issue for their clinical application. Furthermore, the development of neoantigen-centered individual therapies requires a highly customized and personalized approach, which is costly to prepare and poses a serious burden to the families of most oncology patients. Additionally, there is the risk of predictive failures leading to “ineffective” vaccines. Therefore, addressing the time and cost issues associated with neoantigen-based therapies will be paramount to their widespread introduction in lung cancer treatment [57,94,124,192,196,197,198].

### 5.6. Neoantigen Prognostic Monitoring

Both the quantity and immunogenicity of neoantigens play vital roles in shaping the response and prognosis of cancer patients [199]. For instance, in patients with advanced NSCLC following treatment with PD-1 inhibitor (pembrolizumab), exome sequencing revealed that those with higher tumor neoantigen burdens and lower intra-tumor heterogeneity exhibit elevated PD-L1 expression, heightened sensitivity to immune checkpoint inhibitory therapies, and extended overall survival [162,200,201]. Despite these advancements, the assessment of therapeutic vaccine efficacy often relies on indirect indicators. For instance, cytokine secretion only indirectly reflects the generation and effectiveness of the immune response. Consequently, there is a notable absence of direct immunologic effectors and immune response biomarkers capable of accurately indicating clinical efficacy in monitoring vaccine-induced immune responses. Hence, the development of a precise and reliable prognostic monitoring program stands as a pivotal determinant in guiding subsequent treatment strategies for patients.

### 5.7. Drug Resistance

Neoantigen-based therapies hold substantial potential to overcome targeted therapy resistance in lung cancer. By precisely targeting stable clonal mutations, reversing immune desertification, and repairing antigen presentation defects, they serve as a powerful tool against targeted drug resistance. Research indicates that patients with resistant lung cancer often exhibit loss of driver gene mutations (e.g., EGFR ex19del deletion) or subclonal heterogeneity. However, clonal neoantigens—particularly those arising before whole-genome doubling (WGD) during early tumorigenesis—remain stable during metastasis, reducing antigen loss risk from chromosomal instability. This stability enables the induction of specific TCR clones, thereby activating immune responses in NSCLC patients [202]. Furthermore, neoantigen-based approaches remodel the immunosuppressive tumor microenvironment. Tumors resistant to targeted therapies frequently display an “immune-cold” phenotype, characterized by low T cell infiltration and high immunosuppressive cell presence. Neoantigen vaccines counteract this by activating naïve T cells, increasing TILs, and reversing immunosuppression. For instance, Juanita Lopez et al. demonstrated that personalized mRNA vaccines (e.g., Autogene Cevumeran) induced multi-neoantigen-specific T cells in 71% of advanced solid tumor patients, achieving 7.2% intratumoral T cell infiltration. This study also revealed that tumor-infiltrating T cells predominantly exhibited an effector memory phenotype (CD45RO+CCR7−), conferring long-term immune surveillance capabilities [203]. Concurrently, research by Mitchell Emmers and colleagues revealed that 25% to 80% of lung cancers harbor TAP (transporter associated with antigen processing) defects. These defects impair conventional antigen presentation and facilitate immune escape, thereby driving resistance to PD-1/PD-L1 inhibitors. Notably, TEIPP vaccines targeting alternative antigens (e.g., LRPAP1) exposed to TAP-deficient cells could still elicit specific CD8^+^ T cell responses in PD-1-resistant patients (83% response rate). Combining these vaccines with PD-1 inhibitors further expanded T cell populations and enhanced anti-tumor efficacy [204]. Despite offering therapeutic promise, neoantigen immunotherapies face intrinsic resistance mechanisms including: (1) clonal loss of tumor-reactive T cells and T cell exhaustion in immunosuppressive microenvironments, diminishing infused TIL efficacy; (2) neoantigen escape via immunoediting through HLA loss or interferon signaling mutations; and (3) impaired antigen presentation due to epigenetic silencing of transposable elements or MHC-I downregulation [205,206,207]. Future research will focus on systematically addressing these limitations through the integration of tumor genomics, microenvironment analysis, and combination drug strategies.

## 6. Future Prospect and Research Direction of Neoantigens

With the advancement of our understanding of tumor-immune interactions, neoantigen-based therapy, either alone or in combination with other treatment approaches, is undergoing validation in numerous clinical trials. Tumor neoantigen immunotherapy holds promise as a novel therapeutic strategy and a new era of precision therapy. However, challenges and issues still need to be addressed and optimized, particularly in the context of lung cancer, including advanced NSCLC. The rapid and diverse tumor-specific mutations present a challenge in identifying immunological targets [208,209]. Consequently, exploring this aspect will be a key focus of neoantigen research in the future. Several strategies can contribute to the improvement and optimization of neoantigen-based therapy, such as: (1) standardizing and unifying neoantigen prediction algorithms. Incorporating factors such as gene fusion, deletion, and insertion into these algorithms and increasing the proportion of neoantigens of MHC-II molecules can improve the efficiency and accuracy of prediction. The integration of informatics and artificial intelligence will further aid in these efforts. (2) Development of tumor vaccines targeting generic neoantigens generated by driver gene mutations. By focusing on common neoantigens produced by specific gene mutations, it becomes possible to expedite vaccine preparation, reduce costs, and provide rapid and effective treatment to patients with these gene mutations. This approach ensures efficient treatment while advancing the goal of precision medicine. (3) Monitoring treatment progress and assessing clinical efficacy. Regular monitoring of tumor size, recurrence, and changes in the number of neoantigen-reactive T lymphocytes and memory T cells of neoantigenic origin in the patient’s blood before and after treatment can provide valuable insights. Additionally, monitoring changes in the levels of inflammatory molecules like interferon-gamma in the blood can help identify more accurate indicators for assessing clinical efficacy. (4) Overcoming immune escape and tolerance. Single neoantigen therapies may lead to tumor immune escape, immune tolerance, and tumor recurrence. To address this, employing multi-epitope vaccines or combining neoantigen therapy with adjuvants, radiotherapy, and other treatment protocols can broaden the immune response and improve treatment outcomes. With the continuous deepening of neoantigen prediction research on lung cancer and the accumulation of validation databases, we can optimize and enhance the efficacy of neoantigen-based immunotherapy, ultimately improving patient outcomes and paving the way for more effective and precise cancer treatments.

## 7. Conclusions

Lung cancer persists as a principal contributor to global cancer mortality, underscoring the urgent need for transformative therapeutic approaches. Neoantigen-based immunotherapy leveraging tumor-specific antigens derived from somatic mutations offers high specificity and reduced off-target toxicity compared to traditional tumor-associated antigen approaches. Advances in sequencing and bioinformatics enable precise neoantigen prediction, while strategies like personalized vaccines, adoptive cell therapy, and combination regimens with immune checkpoint inhibitors show promising clinical efficacy. However, challenges persist, including tumor heterogeneity, HLA diversity, high costs, and prolonged vaccine preparation. Future efforts should focus on standardizing prediction algorithms, developing shared neoantigen platforms, enhancing immune monitoring, and overcoming immune escape mechanisms. By bridging these scientific advancements with clinical work, neoantigen immunotherapy will deliver sustained benefits for lung cancer patients.

## Figures and Tables

**Table 1:** Comprehensive comparison of neoantigen anti-tumor vaccines.

Vaccine Type	Advantages	Challenges	Differentiations	**Common Features**
Peptide/Protein vaccines	Direct antigen presentation Customizable for MHC-I (short peptides) or MHC-II (long peptides)	Low stability (rapid degradation) Limited immunogenicity	Production complexity: Very High Cost: Very High Target cohort: Individualized	1. Activate tumor-specific T cell immunity against neoantigens. 2. Rely on genomic sequencing and bioinformatic neoantigen prediction. 3. Most show enhanced efficacy with ICIs or conventional therapies. 4. Ranging from fully individualized (DC/peptide vaccines) to semi-/non-personalized (shared/viral vector vaccines).
DC vaccines	High antigen-presenting efficiency Induces long-term immune memory	Complex/costly production Requires strict personalized protocols	Production complexity: Low Cost: Medium Target cohort: Semi-personalized	
Nucleic acid vaccines	Rapid manufacturing Multi-epitope encoding mRNA avoids insertional mutagenesis risks	Low DNA transfection efficiency mRNA requires cold-chain storage	Production complexity: Low Cost: Low Target cohort: Broad (mutation-dependent)	
Viral vector vaccines	High gene delivery efficiency Intrinsic adjuvant effect	Preexisting immunity reduces efficacy Vector-related toxicity risks	Production complexity: Medium Cost: Medium Target cohort: Broad (if no pre-immunity)	
Fusion protein vaccines	Boosts immunogenicity Modulates specific immune pathways	Complex design/engineering Risk of uncontrolled inflammation	Production complexity: High Cost: High Target cohort: Pathway-specific	
Shared neoantigen vaccines	No full personalization needed Low-cost/scalable production	Limited eligible patients Suboptimal immunogenicity in some cases	Production complexity: LowCost: LowTarget cohort: Broad (mutation-dependent)	

Abbreviations: DC, dendritic cell; ICIs, immune checkpoint inhibitors; MHC: major histocompatibility complex.

**Table 2:** Representative clinical studies of neoantigen immunotherapy in lung cancer.

Trail Number	Therapy Type	Tumor	Intervention	Adjuvant Therapy	**Stage**	**Patients**	**Status**
NCT04397926	Vaccine	NSCLC	Individualized neoantigen peptides vaccine	/	Phase 1	20	Unknown status
NCT04266730	Vaccine	Lung cancer	PANDA-VAC(Peptide vaccine)	Pembrolizumab	Phase 1	6	Not yet recruiting
NCT05269381	Vaccine	Lung Cancer	Neoantigen Peptide Vaccine	Pembrolizumab	Phase 1	36	Recruiting
NCT06095934	Vaccine	NSCLC	Neoantigen Peptide Vaccine	/	/	20	Recruiting
NCT06751901	Vaccine	Advanced NSCLC	Neoantigen-based peptide vaccine	Radiotherapy PD-1 inhibitor	Phase 2	10	Recruiting
NCT05098210	Vaccine	Stage III-IV NSCLC	Neoantigen Peptide Vaccine	Nivolumab Poly ICLC	Phase 1	25	Recruiting
NCT04397003	Vaccine	Extensive-stage SCLC	Neoantigen DNA Vaccine	Durvalumab	Phase 2	27	Recruiting
NCT02897765	Vaccine	Lung cancer	NEO-PV-01(DNA Vaccine)	Nivolumab	Phase 1	34	Completed
NCT03380871	Vaccine	NSCLC	NEO-PV-01(DNA Vaccine)	Pembrolizumab Carboplatin Pemetrexed	Phase 1	38	Completed
NCT03908671	Vaccine	NSCLC	Personalized mRNA Tumor Vaccine	/	/	24	Recruiting
NCT03639714	Vaccine	NSCLC	GRT-C901/GRT-C902(mRNA Vaccine)	Nivolumab Ipilimumab	Phase1/2	29	Completed
NCT06735508	Vaccine	NSCLC	mRNA Neoantigen Vaccine	Adebrelimab	Phase1	40	Not yet recruiting
NCT06685653	Vaccine	NSCLC	RGL-270 (mRNA vaccine)	Adebrelimab	Phase 1	65	Not yet recruiting
NCT04078269	Vaccine	NSCLC	MIDRIXNEO-LUNG (DC Vaccine)	/	Phase 1	6	Completed
NCT03871205	Vaccine	Lung cancer	Neoantigen loaded DC Vaccine	/	Phase 1	30	Unknown status
NCT02956551	Vaccine	NSCLC	DC Vaccine	/	Phase 1	20	Unknown status
NCT04147078	Vaccine	NSCLC	DC Vaccine	/	Phase 1	80	Recruiting
NCT06751849	Vaccine	Advanced NSCLC	Neoantigen loaded DC vaccine	Radiotherapy PD-1 inhibitor	Phase 2	10	Recruiting
NCT05886439	Vaccine	Advanced lung carcinoma	LK101(personlized neoantigen pulsed DC Vaccine)	Pembrolizumab; Durvalumab	Phase 1	40	Recruiting
NCT06329908	Vaccine	Neo-DCVac	Lung Cancer	ICIs	Phase 1	20	Recruiting
NCT04487093	Vaccine	NSLCL	neoantigen vaccine	EGFR-TKI Anti-angiogenesis drug	Phase 1	20	Unknown status
NCT03807102	Vaccine	Lung cancer	Neoantigen tumor vaccine	/	Phase1/2	30	Not yet recruiting
NCT04998474	Vaccine	NSCLC	FRAME-001 personalized vaccine	/	Phase 2	15	Unknown status
NCT03633110	Vaccine	NSCLC	GEN-009 Adjuvanted Vaccine	Nivolumab; Pembrolizumab	Phase 1/2	24	Completed
NCT03715985	Vaccine	NSCLC Metastatic	EVAX-01- CAF09b	/	Phase1/2	12	Unknown status
NCT03953235	Vaccine	NSCLC	GRT-C903/GRT-C904	Nivolumab; Ipilimumab	Phase 1/2	39	Completed
NCT03552718	Vaccine	NSCLC	YE-NEO-001	/	Phase 1	16	Active, not recruiting
NCT03205930	Vaccine	NSCLC Stage IV	Neo-MASCT	/	Phase 1/2	20	Unknown status
NCT03289962	Vaccine	NSCLC	RO7198457	Atezolizumab	Phase 1	272	Active, not recruiting
NCT05292859	TCR-T	Squamous cell lung Cancer; Adenocarcinoma of lung; Adenosquamous cell Lung Cancer	Neoantigen specific TCR-T cell drug product	/	/	8	Active, not recruiting
NCT05194735	TCR-T	NSCLC Squamous Cell Lung Cancer; Lung Adenocarcinoma; Adenosquamous cell lung cancer	Neoantigen specific TCR-T cell drug product	/	Phase 1/2	18	Active, not recruiting
NCT03970382	TCR-T	NSCLC	NeoTCR-P1 adoptive cell therapy	Nivolumab	Phase 1	21	Suspended
NCT03412877	TCR-T	NSCLC	Individual Patient TCR-Transduced PBL	Cyclophosphamide Fludarabine Aldesleukin Pembrolizumab (KEYTRUDA)	Phase 2	270	Recruiting
NCT05798533	ACT	NSCLC	Neo-T	Toripalimab; Tislelizumab	Phase 1	6	Unknown status
NCT05798546	ACT	NSCLC	Neo-T	Cyclophosphamide; Fludarabine; Interleukin-2	Phase 1	21	Unknown status

Abbreviations: NSCLC, non-small cell lung cancer; SCLC, small cell lung cancer; DC, dendritic cell; Neo-DCVac, neoantigen-loaded dendritic cell vaccine; ICIs, immune checkpoint inhibitors; TCR-T, T cell receptor modified T cells; ACT, adoptive cell therapy.

## Data Availability

All images included in the review are property of the authors.

## Abbreviations

IARC	International Agency for Research on Cancer
SCLC	small cell lung cancer
NSCLC	non-small cell lung cancer
ICI	immune checkpoint inhibitor
TSA	tumor-specific antigen
TAA	tumor associated antigen
SNV	single nucleotide variant
INDELS	insertions/deletions
MHC	major histocompatibility complex
PTM	post-translational modification
APC	antigen-presenting cell
DC	dendritic cell
TDLN	tumor draining lymph node
TCR	T cell receptor
NGS	next-generation sequencing
WGS	whole genome sequencing
WES	whole exome sequencing
RNA-seq	RNA sequencing
HLA	human leukocyte antigen
SHERPA	systematic hla epitope ranking pan algorithm
TAP	tumor abnormal protein
CNN	convolutional neural network
SABR	signaling and antigen-presenting bifunctional receptor
MCR	MHC-TCR chimeric receptor
ACT	adoptive cell therapy
TCR-T	T cell receptor modified T cell
TIL	tumor-infiltrating lymphocyte
PFS	progression-free survival
OS	overall survival
WTL	whole tumor lysate
CEA	carcinoembryonic antigen
IFN-γ	interferon-γ
IL-2	interleukin-2
PEV	personalized vaccine
CAR-T	chimeric antigen receptor modified T cells
PFS	progression-free survival
TCRm	TCR mimics
MANA	mutation-associated neoantigen
ADCs	antibody-drug conjugates
BsAbs	bispecific antibodies
scFvs	single-stranded variable fragments
scDbs	single-stranded disomes
TME	tumor microenvironment
SD	stable disease
PR	partial response
ctDNA	circulating tumor DNA
Treg	T regulatory
